# Alcohol and Psychological Predictors of Daily Functioning by Sexual Minority Status in Older Adults

**DOI:** 10.1093/geroni/igaf122.3702

**Published:** 2025-12-31

**Authors:** Carolina L Costa, Carlos Araujo-Menendez, Ariana Stickel

**Affiliations:** San Diego State University, San Diego, California, United States; San Diego State University/University of California, San Diego, San Diego, California, United States; San Diego State University, San Diego, California, United States

## Abstract

Sexual minority (SM; e.g., lesbian, gay, bisexual, or queer+) adults are at increased risk of poor physical and mental health outcomes, likely due to discrimination and related stress. Stress may also drive health behaviors, including increased alcohol use. Therefore, we examined if alcohol use interacts with psychological factors to predict activity of daily living (ADL) limitations among SM and non-SM older adults. Participants included 5,643 adults (SM = 321; M = 72-years, SD = 10-years) from the 2022 Health and Retirement Study. Three-month alcohol use was assessed through five self-reported items (ever drank, ever drank more than 12+ drinks, drinks per week, drinks per day, binges). ADL limitation (yes/no) was defined as 1+ self-reported difficulties across five items. Psychological factors included depressive symptoms (CESD-8) and loneliness (yes/no; 1 item). SM-status-stratified logistic regression models were performed to assess the moderating effect of alcohol in the association between psychological factors and ADL limitations while adjusting for sociodemographic variables. In a model with non-SM adults, higher depression scores were associated with greater odds of ADL limitations, especially for non-drinkers compared to drinkers (OR = 0.90, 95% CI [0.82, 0.97]). In a model with SM adults, endorsing loneliness was associated with more ADL limitations at higher levels of number of drinks per day (OR = 4.45, 95% CI [1.39, 18.55]). These differences may reflect how alcohol use interacts with mental health in distinct ways for SM and non-SM older adults.

